# Essential Oils as Multicomponent Mixtures and Their Potential for Human Health and Well-Being

**DOI:** 10.3389/fphar.2022.956541

**Published:** 2022-08-24

**Authors:** Marek Bunse, Rolf Daniels, Carsten Gründemann, Jörg Heilmann, Dietmar R. Kammerer, Michael Keusgen, Ulrike Lindequist, Matthias F. Melzig, Gertrud E. Morlock, Hartwig Schulz, Ralf Schweiggert, Meinhard Simon, Florian C. Stintzing, Michael Wink

**Affiliations:** ^1^ Department of Analytical Development and Research, WALA Heilmittel GmbH, Bad Boll, Germany; ^2^ Department of Pharmaceutical Technology, University of Tübingen, Tübingen, Germany; ^3^ Translational Complementary Medicine, Department of Pharmaceutical Sciences, University of Basel, Basel, Switzerland; ^4^ Department of Pharmaceutical Biology, University of Regensburg, Regensburg, Germany; ^5^ Institute of Pharmaceutical Chemistry, Philipps-Universität Marburg, Marburg, Germany; ^6^ Institute of Pharmacy, Ernst-Moritz-Arndt-University Greifswald, Greifswald, Germany; ^7^ Institute of Pharmacy, Freie Universität Berlin, Berlin, Germany; ^8^ Institute of Nutritional Science, Chair of Food Science and TransMIT Center for Effect-Directed Analysis, Justus Liebig University Giessen, Giessen, Germany; ^9^ Consulting & Project Management for Medicinal & Aromatic Plants, Stahnsdorf, Germany; ^10^ Institute of Beverage Research, Chair of Analysis and Technology of Plant-Based Foods, Geisenheim University, Geisenheim, Germany; ^11^ Institute for Chemistry and Biology of the Marine Environment, University of Oldenburg, Oldenburg, Germany; ^12^ Institute of Pharmacy and Molecular Biotechnology, Heidelberg University, Heidelberg, Germany

**Keywords:** essentail oils, multicomponent mixtures, integrative medicine, phytotherapy, antibiotic resistance

## Abstract

Essential oils (EOs) and their individual volatile organic constituents have been an inherent part of our civilization for thousands of years. They are widely used as fragrances in perfumes and cosmetics and contribute to a healthy diet, but also act as active ingredients of pharmaceutical products. Their antibacterial, antiviral, and anti-inflammatory properties have qualified EOs early on for both, the causal and symptomatic therapy of a number of diseases, but also for prevention. Obtained from natural, mostly plant materials, EOs constitute a typical example of a multicomponent mixture (more than one constituent substances, MOCS) with up to several hundreds of individual compounds, which in a sophisticated composition make up the property of a particular complete EO. The integrative use of EOs as MOCS will play a major role in human and veterinary medicine now and in the future and is already widely used in some cases, *e.g.*, in aromatherapy for the treatment of psychosomatic complaints, for inhalation in the treatment of respiratory diseases, or topically administered to manage adverse skin diseases. The diversity of molecules with different functionalities exhibits a broad range of multiple physical and chemical properties, which are the base of their multi-target activity as opposed to single isolated compounds. Whether and how such a broad-spectrum effect is reflected in natural mixtures and which kind of pharmacological potential they provide will be considered in the context of ONE Health in more detail in this review.

## History of Phytotherapy

Aromatic plants have long been used in traditional medicine for their protective and therapeutic properties, in foods to impart flavor, but also as anti-inflammatory, antioxidant and antimicrobial agents (Freitas and Cattelan, 2018).

In Europe, phytotherapy is the best-known field of natural medicine today. One of the first and most detailed pharmacognostic guides on plants but also animals and their compounds such as essential oils or fatty acids is Dioscorides’ ʻDe Materia Medicaʼ (first century) (Staub et al., 2016). In mediaeval times (5^th^–15^th^ century) herbs and extracts prepared therefrom were used for the treatment of various diseases, especially in monasteries, but also by healers, mostly women, who knew the potential of such preparations and later (since 13^th^ century) by monks and pharmacists (Dufault et al., 2001). In the age of the plague (14^th^ century), numerous epidemics of bubonic plague and other infectious diseases killed about 25% of the European population, which in consequence led to massive restrictions and regression in all areas of life. Despite basic knowledge of herbalism, nursing and medicine, the health system of mediaeval times could not prevent the consequences of the pandemic. At that time, living and working conditions as well as famines due to social structures and failed harvests were responsible for the fact that people were not able to live under adequate hygienic conditions, let alone to secure nutrition.

With the discovery of America and the sea route to India at the end of the 15^th^ century (Renaissance), Europeans discovered plants that were previously unknown to them (e.g., cacao, chili pepper, sunflower), as well as options to treat further kinds of ailments. Thanks to printing, medicinal plants could be depicted and not only described, thus, they were more broadly recognized and knowledge could be shared to a greater extent. The most popular herbaria of this period are the “Herbarium Vivae Eicones” (1530) by Otto Brunfels, the “De Historia Stirpium” (1542) by Leonhart Fuchs, the “Herbarium” (1597) by John Gerard, the “English physician” (1649) by Nicholas Culpeper, and the “Theatrum botanicum” (1669) by John Parkinson (Makarska-Białokoz, 2020). A real breakthrough in the history of herbal medicine was initiated by Paracelsus, and especially his most famous phrase in relation to toxicology “Poison is in everything, and no thing is without poison; the dose makes it either a poison or a remedy” (Philippus Theophrastus Aureolus Bombastus von Hohenheim, 1493–1541; Holzinger, 2013). He studied the signature of plants, developed methods for extracting “therapeutic essences” from medicinal plants, and therefore is regarded as the father of phytochemistry and pharmacognosy.

Beginning in the 18^th^ and 19^th^ centuries, people have been increasingly concerned by the chemical knowledge of herbs and their constituents as well as their possible effects on health. Since then, it had been believed that individual specific chemical compounds are responsible for the healing properties of a medicinal plant and that effective preparations should consist of standardized, easily dosable substances or extracts. For this reason, individual constituents began to be increasingly isolated from plants. At that time, morphine isolated from opium poppy was considered a breakthrough in the study of medicinal plants by the pharmacist Friedrich Wilhelm Adam Sertürner (Sertürner, 1806). Worldwide, many further important compounds were isolated from plants, such as strychnine, quinine, caffeine, salicin, cocaine, and digitalin, to name a few (Barnes, 2007). The era of scientific research on the chemical profile, the pharmacological and toxicological properties of plant extracts had dawned. Favored by the development of novel chromatographic and microscopic methods, herbs became the sources of medicines in the 20^th^ century. Until 1930, herbal medicines were very popular and only gradually replaced by preparations obtained from chemical synthesis, the basis of synthetic drugs (Ferreira et al., 2014; Jamshidi-Kia et al., 2018; Makarska-Białokoz, 2020).

The trend of isolating single compounds was countered by the development of scientifically based rational phytotherapy. Distinct cultivation and processing methods were the basis of new guidelines, such as the World Health Organization (WHO) Guidelines on Good Agricultural and Collection Practices (GACP) for Medicinal Plants or the European Medicines Agency (EMA) Guideline on Quality of Herbal Medicinal Products (CPMP/QWP/2819/00), herbal medicines of tested quality were developed from then on (Sahoo et al., 2010; Fürst and Zündorf, 2015). Today it seems to become more important than ever to develop effective and clinically proven, but also affordable medicines including traditional, long-established plant preparations. Furthermore, medicinal plants continue to hold great promise providing anti-inflammatory, antibacterial, antifungal, antiviral, anticancer, and antiparasitic compounds as leads. Thanks to their evolutionary development, plants possess a quite comprehensive arsenal of defense, protection, distribution and attraction strategies (Wink, 2003; Sakkas and Papadopoulou, 2017) based on bioactive and potentially pharmacologically active specialized metabolites. Given this considerable potential, medicinal plant extracts and their complex pharmacological and physiological effects are still highly underexplored, and the awareness of the need for more sound scientific studies is growing to better understand the rationale and the underlying principles of traditional therapeutic uses.

## Inherent Complexity

The basis of the diverse biological properties of medicinal plants and extracts therefrom is the interplay of complex secondary constituents such as alkaloids, terpenes, flavonoids, tannins, etc., as well as non-coding small RNA species, such as microRNA (Xie et al., 2016), which explain their diverse pharmacological and therapeutic properties both as individual compounds and as complex mixtures (MOCS; Wink, 2015). The latter are preparations used in phytotherapy, but which may also be applied as adjunctive therapy to a single compound of either synthetic or natural origin in the context of integrative medicine. Individual constituents in natural MOCS are often present at lower levels, compared to the amounts used in therapy with isolated individual components (Rasoanaivo et al., 2011; Gorlenko et al., 2020). Interestingly, a neat substance at the same level as present in natural plant source mixtures, usually does not reach the same pharmacological effects on a quantitative and qualitative scale. This means that the same chemical constituent may exhibit a clear biological activity when forming part of a natural mixture (*e.g.*, a plant extract), whereas the isolated compound may not. Thus, natural compounds present in combination may enhance each other (synergism), complement each other (additive effect), or attenuate each other (antagonism) (Chou, 2006; Hemaiswarya et al., 2008; Połeć et al., 2022). This complex interplay of substances responsible for such effects may not only exhibit positive effects, but may also compensate for, attenuate or cancel out possible undesirable effects of other components (Caesar and Cech, 2019). The recent combination of planar chromatography with multiplex biological effect detection can straightforwardly differentiate such effects on the same plate (Ronzheimer et al., 2022; Schreiner et al., 2022). In addition, unlike isolated individual substances, MOCS often exhibit a broad spectrum of action (*i.e*., they are multi-target drugs), since the diversity of molecular structures (multi-component), with their specific functional moieties and their respective chemical and physiological properties do not only have one common target, *i.e*. the “multi components result in multi targets” theory (Nahrstedt and Butterweck, 2010; Schwabl et al., 2013). The streamlined analysis of 68 different botanicals does not only highlight such versatility in potential biofunctional properties, but also prove that such multi-compounds may interact with various metabolic pathways (Morlock, 2021; Schreiner et al., 2021). Furthermore, MOCS may modulate their mutual resorption, but also for specific compounds from food or medicines and may also affect typical characteristics of smell and taste (Karalis et al., 2008; Ku et al., 2020).

Nevertheless, there is a strong trend in health policy and the pharmaceutical industry to replace complex natural MOCS, such as EOs, with isolated mono-substances in the future. The main reasons to prefer mono-substances may be simpler quality control, better application and standardization, straightforward clinical studies, with known active targets including side effects. In addition, authenticity control of EOs may be a major issue. By means of a broad portfolio of methods, like chiral gas chromatography, isotope-ratio mass spectrometry, NMR, thin-layer chromatography, vibrational spectroscopy, multi-dimensional chromatography, high-performance liquid chromatography, headspace chromatography, and combination with chemometrics-metabolomics, adulterated or synthetic oils may be identified (Do et al., 2015). The more complex a MOCS is, the more difficult it seems to verify its authenticity and genuineness. This seems to be another reason why biologically active mono-substances are often favored. When mono-substances are used, the potential of synergistic action is lost, which has recently been discovered in a number of natural MOCS (Ronzheimer et al., 2022; Schreiner et al., 2022). However, rising costs for mono-compound isolation and purification increasingly question the positive economic balance, especially in light of the growing awareness for the ecological impact and its ramifications. In addition, patient-centered medicine asks for complementary approaches as a companion to conventional mono-substance therapies (Agarwal, 2018; Clark et al., 2021) and other effects and therapeutic benefits with reduced side effects can be achieved by applying MOCS compared to single compound application. Therefore, there is an urgent need to re-evaluate MOCS, and support sound studies to discover their full potential for human but also animal health in the context of integrative health approaches (Agarwal, 2018; Clark et al., 2021). This should go along with sustainable cultivation, not only to maintain and create jobs and to meet the demand for medicinal plants, but also to cultivate medicinal plants in an environmentally conscious manner, to preserve protected species and to protect wild stocks from uncontrolled collection (Silori and Badola, 2000; Akinyemi et al., 2018).

## Essential Oils As Classical MOCS

Essential oils (EOs) are among the most versatile and long-term used medicinal plant preparations (Plant et al., 2019). They are produced in more than 17,500 aromatic species and are stored in various plant organs, *i.e*., blossoms (*e.g*., *Rosa* x *damascena* Herrm. (Rosaceae)*,* damask rose), leaves (*e.g*., *Cymbopogon citratus* (DC.) Stapf (Poaceae), lemon grass), wood (*e.g*., *Santalum acuminatum* (R.Br.) A.DC. (Santalaceae), sandalwood), roots (*e.g*., *Chrysopogon zizanioides* L. (Poaceae), vetiver), rhizomes (*Zingiber officinale* Roscoe (Zingiberaceae), ginger; *Curcuma longa* L. (Zingiberaceae), turmeric), fruits (*e.g*., *Pimpinella anisum* L. (Apiaceae), anise and *Carum carvi* L. (Apiaceae), caraway) (Regnault-Roger et al., 2012). Essential oils are defined as mixtures of secondary metabolites from plants (Ahmad et al., 2021) and typically exhibit a strong odor as they are MOCS containing a variety of volatile terpenes, aldehydes, alcohols, ketones and simple phenolics (Bakkali et al., 2008; Sadgrove et al., 2022). The European Chemicals Agency (ECHA) has defined EOs as “a volatile part of a natural product obtained by distillation, steam distillation or, in the case of citrus fruits, by squeezing. It contains mainly volatile hydrocarbons. Essential oils are derived from various parts of plants.” (Essential oils - ECHA, 2022). In the plant, they play a central role in pollination, communication, and protection: They attract natural enemies of herbivores, protect against pathogens such as fungi and bacteria, are messengers between plants, attract seed dispersers and particularly pollinators, protect against extreme temperature fluctuations, etc. (Holopainen, 2004; Dudareva et al., 2006; Loreto et al., 2009; Loreto and D'Auria, 2022). As natural mixtures and the products obtained therefrom, EOs can vary in quality, quantity, and composition even when obtained from the same plant species. Not only the choice of the EO recovery process, but also the plant organ used and exogenous factors during plant growth such as climate, soil conditions, pest infestation, age and stage of the vegetation cycle play a decisive role (Masotti et al., 2003; Angioni et al., 2006). In addition, the composition of EOs may differ between individual plants in a population and sometimes even different chemotypes exist within a species. Furthermore, chemical varieties of EO-producing plants have also been bred, developed, and studied to improve their EOs content, quality and composition (Toxopeus and Bouwmeester, 1992; Sarrou et al., 2017; Lal et al., 2018).

## Biosynthesis and Chemical Composition

**FIGURE 1:**
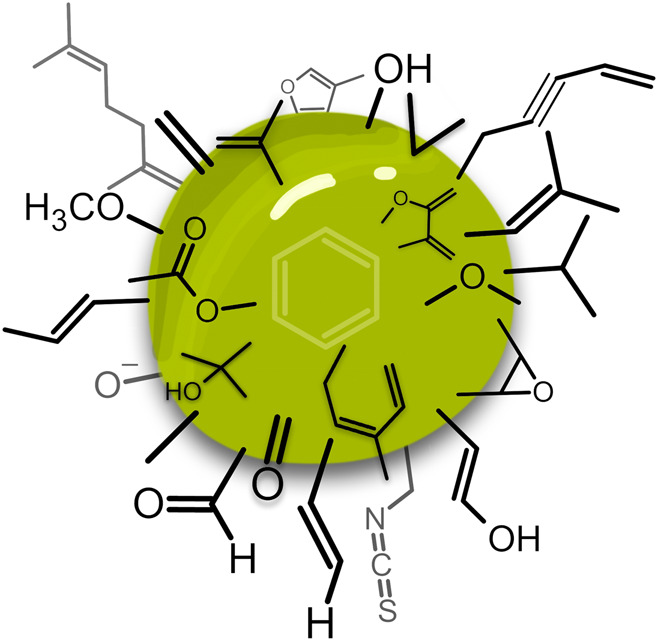
Concept of EOs acting as MOCS with multi-target functional groups.

**SCHEME 1:**
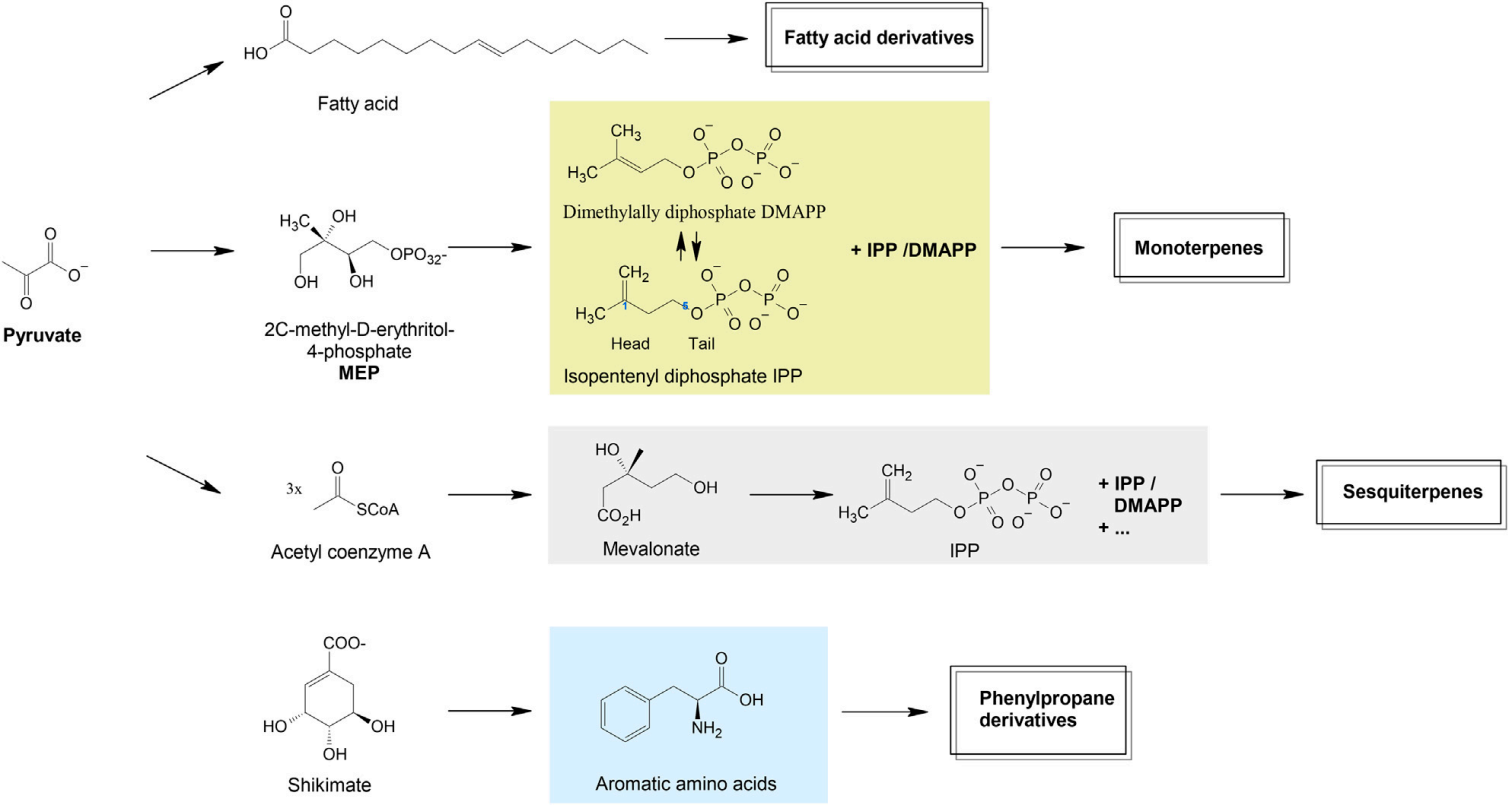
Biosynthetic pathways of major volatile organic compounds. Modified according to Caissard et al. (2004).

In aromatic and scented plants, the vast majority of volatile organic compounds originate from three precursor categories (Figure 1), namely phenolic compounds from shikimate and acetate malonate pathway, fatty acid derivatives, and isoprenoids (Sangwan et al., 2001; Caissard et al., 2004). The main constituents of EOs are often isoprenoids, which form the core structures of terpenoids (Scheme 1). The basic structure of isoprenoids consists of 2-methylbutane moieties (isoprene or 2-methylbutadiene units), which are biosynthesized in the cytosol via the mevalonic acid pathway and/or in plastids *via* the 2-C-methylerythritol-4-phosphate (MEP) pathway (Newman and Chappell, 1999). The universal precursors of all terpenoids are the active isoprene unit, isopentenyl diphosphate (IPP), and its isomer dimethylallyl diphosphate (DMAPP). Monoterpenoids are usually formed by the fusion of IPP in a head-to-tail manner to its isomer DMAPP, leading to geranyl diphosphate (GPP) (Dubey et al., 2003). In case of sesquiterpenoids GPP will be further head-to-tail elongated by a second IPP to the C15 farnesyl diphosphate.

The different biosynthetic pathways of EO substances constitute the fundament of their structural diversity. Beside terpenes another main group of EOs is composed of aromatic and aliphatic constituents.

A common analytical method for assessing commercially available EOs is chemical characterization by gas-liquid-chromatography coupled to mass spectrometry. In addition, analytical techniques such as nuclear magnetic resonance (NMR) spectroscopy and headspace gas chromatography play a role in characterizing and evaluating EOs quality and authenticity, especially for unstable EO molecules like thermolabile sesquiterpenes, such as from *Curcuma caesia* Roxb. (Zingiberaceae) rhizome (Fakhari et al., 2005; Turek and Stintzing, 2011; Mahanta et al., 2020; Truzzi et al., 2021). According to Bakkali et al. (2008), about 3,000 EOs are known to date from a wide variety of plants and their organs, of which 300–400 are particularly important in the medicinal, pharmaceutical, agricultural, food, sanitary, cosmetics and perfume industries, as well as in dentistry, as adhesives and flavors (Ramezani et al., 2008; Turek and Stintzing, 2013; Ibrahim, 2020).

EOs are MOCS normally consisting of 20–200 single compounds, which divide into main constituents with concentrations ranging from 20 to 95%, minor compounds (1%–20%) and trace compounds (<1%) (Stahl-Biskup and Reher, 1987). The specific compound fingerprint is a result of multiple factors, such as plant species, plant part, growing conditions and time of harvest. In most cases, predominant constituents with low odor thresholds determine the typical olfactory EO character. For example, the quantitatively dominating compounds of oregano oil (*Origanum vulgare* ‘Compactum’ L.; Lamiaceae) are carvacrol (30%–80%) and thymol (27%–80%), whereas in fresh oregano leaves the main fragrance components are *γ*-terpinene, *p*-cymene, thymol, and carvacrol (Asadollahi-Baboli and Aghakhani, 2015). In coriander oil (*Coriandrum sativum* L.; Apiaceae) linalool (68%) dominates. In white wormwood oil (*Artemisia herba-alba* Asso; Asteraceae): *α*- and *β*-thujone (57%) and camphor (24%) are the quantitively dominating compounds. For camphor tree oil (*Cinnamomum camphora* (L.) J. Presl; Lauraceae), it is D-camphor (50%), for dill oil (*Anethum graveolens* L.; Apiaceae) *α*-phellandrene (up to 32%) and limonene (up to 32%) from leaf and carvone (up to 55%) and limonene (up to 45%) from the fruit. Peppermint oil (*Mentha* x *piperita* L.; Lamiaceae) is characterized by high amounts of menthol (up to 45%) and menthone (up to 15%) (Beigi et al., 2018; Bhavaniramya et al., 2019; Dobreva and Dimov, 2021; Poudel et al., 2021).

However, also minor or trace components may produce intense odors, which contribute to the characteristic flavor (Góra and Brud, 1983). For example, the fragrance of damask rose (*R. damascena*) is characterized by about 27 main compounds. However, only a few compounds (*i.e*., *β*-damascenone, rose oxide, *trans*-nerolidol, rotundone, 4-(4-methylpent-3-en-1-yl)-2(5H)-furanone) represented by less than 1%, contribute to the distinctive scent of rose oil and account for about 90% of the odor content due to their low odor threshold (Naquvi et al., 2014; Ohashi et al., 2019). This means that not only a quantitative evaluation of compounds but also a qualitative view is required to reveal the full potential of EOs as a classical MOCS.

## Production of EOs

Various methods to obtain EOs comprise conventional and modern techniques, including water or steam distillation, solvent extraction, expression, extraction with supercritical fluids and subcritical water (Edris, 2007). The focus of novel environmentally friendly extraction technologies is to minimize the use of solvents while producing high-quality extracts in a more cost-efficient and process-optimized manner (Chemat et al., 2012). Hence, in addition to biotic and abiotic factors, reproducible and uniform extraction procedures play an important role in achieving consistent quality and composition of EOs. A reliable repertoire of methods exist to control and guarantee their quality, safety and efficacy (Bakkali et al., 2008).

Hydrodistillation, the boiling of plant material in water or the treatment of plant material by steam, is the predominant (historical) method to produce EOs. Nowadays, steam distillation is common for the recovery of most EOs. Hydrodistillation is a softer technique than dry distillation, normally used for wood and bark, because the plant components are exposed to lower temperatures to recover volatiles, and thus thermal decomposition of individual constituents and the production of artefacts in this process is reduced (Aziz et al., 2018).

Ideally, the EO should be distilled from a single species without removing or adding individual EO components. However, not all EOs meet these criteria; for example camphor oil and ylang-ylang oil are fractionated and corn mint is dementholized (Chen et al., 2013). In addition, low recoveries may be opposed to EO production from one plant species, which is why sometimes equivalent species are used, as in the case of anise EOs (Sultanbawa, 2016; Shahrajabian et al., 2019). Furthermore, in some cases it cannot be completely ruled out that another plant species of equal value also is harvested, which might be the case with spruce needle oils (Metsämuuronen and Sirén, 2019; Mofikoya et al., 2022). The chemical composition of EOs is not necessarily identical to that found in the respective living plant. Often, very high-boiling or low-boiling volatile plant compounds are simply lost because they do not even enter the vapor phase or readily evaporate and thereby escape during the production process. While most components shall be retained upon distillation, others may undergo chemical changes; such as the formation of chamazulene from matricin in chamomile (Ramadan et al., 2006). Thus, the composition of EOs represents the final image including the respective biosynthetic fingerprint but also the modified substances due to preceding processing. Furthermore, in some cases, individual compounds are intentionally removed because of their toxicity, such as hydrogen cyanide from bitter almond oil or methyl eugenol from rose oil (Rusanov et al., 2012; Zhang et al., 2019). One important modified oil, *e.g*., by increasing the cineole content, while reducing the acid content, is eucalyptus oil (European Medicines Agency, 2014).

Some EOs may also be recovered by cold pressing. These cold-pressed oils are generally derived from citrus fruits, although distilled citrus oils are also produced. Unprocessed citrus oils may contain non-volatile phototoxic compounds (*i.e*., furocoumarins) that, due to their molecular weight and non-covalent intermolecular binding forces, may remain in cold-pressed but not in distilled citrus oils (Ferhat et al., 2007). In addition, fragrance oils can also be extracted with organic solvents (*n*-hexane), producing concretes, absolutes or resinoids, with liquid or super-critical carbon dioxide, resulting in CO_2_ extracts, or with some innovative methods like ionic liquid, or deep eutectics extraction (Lago et al., 2014; Erşan et al., 2018; Choi and Verpoorte, 2019). A concrete contains both volatile compounds and the non-volatile plant waxes and is prepared by washing the plant material with a non-polar solvent such as *n*-hexane. Absolutes are produced by re-extracting concentrated concretes with ethanol, subjecting to cold temperatures and then the soluble portion is concentrated to obtain fragrances devoid of waxes (Aycı et al., 2005). In addition, the enfleurage process is a very old method of extracting fragrances, in which volatile fragrance molecules from plant parts are transferred to fat in which they are embedded (Shankar et al., 2021).

## EOs and Their Bio-Functional Properties – Risks and Side Effects of Improper Application

**FIGURE 2:**
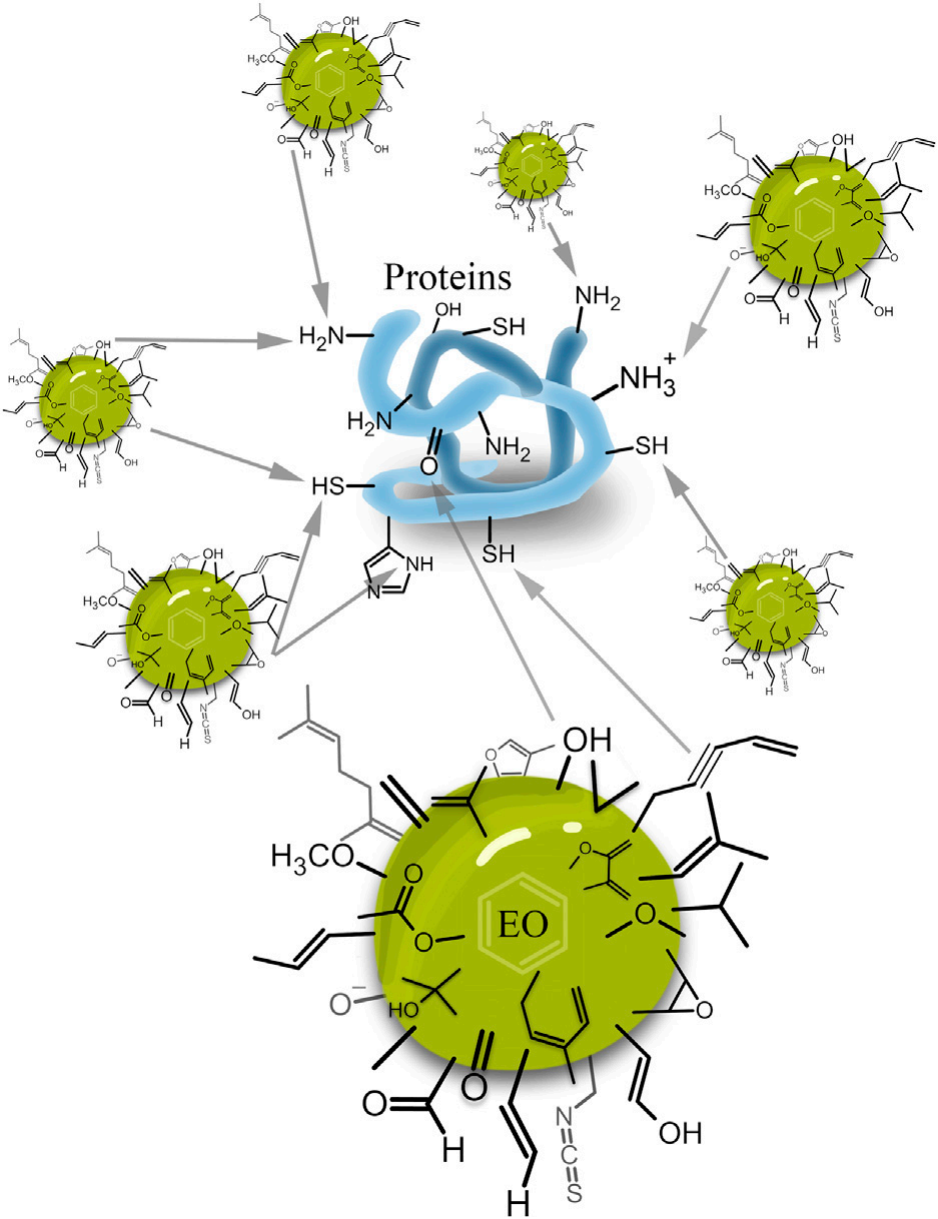
EOs as MOCS and their potential multi-target interactions with proteins.

**FIGURE 3:**
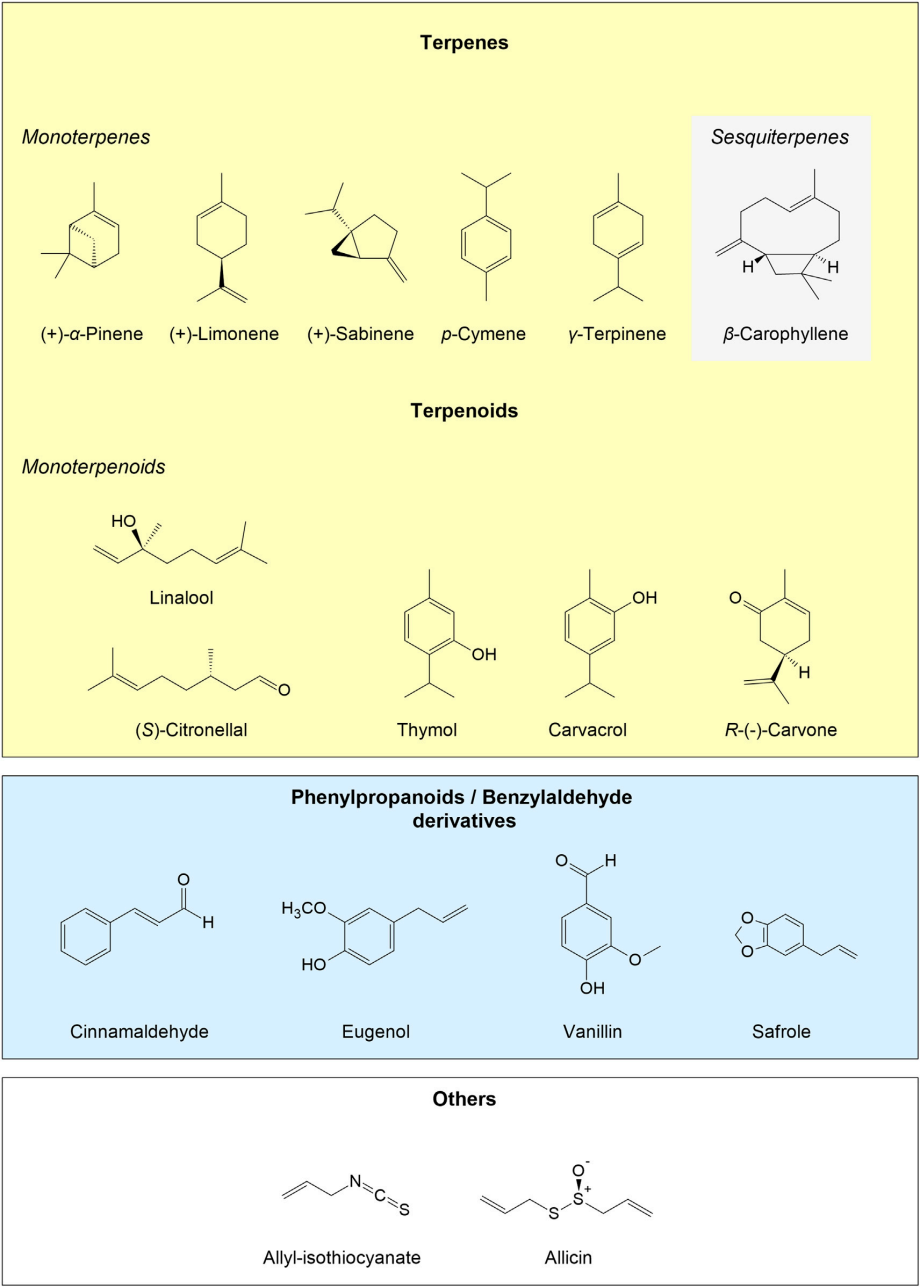
Examples of chemical structures of EO constituents. Modified according to Hyldgaard et al. (2012).

Because of the diverse structural diversity and number of constituents (Figures 1, 2), EOs as a whole do not seem to have selective or singular cellular targets (Carson et al., 2002). Due to their different compound profiles, they can penetrate the cell wall of microorganisms and the cytoplasmic membrane of cells and thus disrupt the structural assembly of saccharides, proteins, fatty acids, and phospholipids, which modulate membrane permeability and fluidity (Figure 3). Such interactions with biomembranes are the basis of nearly all biological activities of EOs and their metabolites to cross cellular compartments. In this way, EOs may cause depolarization of the mitochondrial membranes of eukaryotic cells, by decreasing the membrane potential, impairing the ionic Ca^2+^ cycle and other ionic channels thereby reducing the pH gradient. This may affect crucial metabolic processes such as the proton pump and the ATP (adenosine triphosphate) pool and may encompass cytotoxic activities (Richter and Schlegel, 1993; Novgorodov and Gudz, 1996; Vercesi et al., 1997). EOs may also modify the fluidity of membranes by making them more permeable, thus leading to the leakage of radicals, cytochrome C, calcium and other ions as well as proteins, as in the case of oxidative stress and bioenergetic failure. This may finally lead to cell death by apoptosis and necrosis (Yoon et al., 2000; Armstrong, 2006). Toxicity assessment of a substance or mixture is performed by means of the selectivity index (SI). The SI expresses the ratio between measured cytotoxicity towards normal cells and a desired measured activity, such as antiviral, or anticancerogenic activity. An ideal antiviral or anticancer compound would be cytotoxic against normal cells only at very high concentrations and exhibit antiviral or anticancer activity at very low concentrations (Prayong et al., 2008; Astani et al., 2010; Reichling, 2021). The cytotoxic properties are of great importance for the use of EOs against certain human or animal pathogens, parasites or abnormal cells but undesirable in cosmetic products (Bakkali et al., 2008). Due to their mode of action, acting on multiple targets simultaneously, resistance and adaptation phenomena towards EOs or individual compounds thereof have only scarcely been described. For example, resistance of *Bacillus cereus* to carvacrol has been detected after growth in the presence of a sublethal concentration of this component (Ultee et al., 2000; Di Pasqua et al., 2006). Furthermore, *Pseudomonas aeruginosa* showed increased tolerances to the EO of *Melaleuca alternifolia* (Maiden & Beche) Cheel, which was accompanied by changes in the barrier and energy functions of the outer membrane of the bacterium (Longbottom et al., 2004). This is a worthwhile aspect in finding alternatives to antibiotic therapy where resistance phenomena are increasingly being observed.

In the context of safety evaluation, not only cytotoxicity is relevant, but also mutagenicity and genotoxicity. Antimutagenic properties of an EO (*in vitro*) appear to be based on preventing mutagens from entering the cells, inactivating them by direct scavenging, neutralizing their radical oxygen species (ROS) by binding antioxidant molecules, or activating their own cell-protective antioxidant pathways. In addition, metabolic conversion of promutagens to mutagens can be inhibited by cytochrome P_450_ isoenzymes, or enzymatic processes metabolize harmful mutagens and other xenobiotics to harmless metabolites (Ramel et al., 1986; Kada and Shimoi, 1987; De Flora and Ramel, 1988; Hartman and Shankel, 1990; Kuo et al., 1992; Shankel et al., 1993; Waters et al., 1996; Sharma et al., 2001; Gomes-Carneiro et al., 2005; Ipek et al., 2005). ROS alone may trigger DNA mutation and antioxidants, such as some EO components, may inhibit this process and thus prevent the development of diseases (van Wyk and Wink, 2015). The reduced frequency of mutations caused by EOs was always accompanied by a synergistic induction of complete petite mutants (mitochondrial gene mutations of the respiratory chain). Moreover, EOs alone or in combination with other pharmaceuticals were shown to induce mainly necrosis and not apoptosis. This supports the fact that petite mutants are true rho0 mutants. These are ultimately unable to induce apoptosis due to the lack of functional mitochondria, but only passively induce necrosis (Van Houten et al., 2006).

A number of studies on various EOs and their isolated major constituents have shown that there is no evidence for a nuclear DNA mutation neither from the complete formulation nor the isolated constituents (Bakkali et al., 2005). However, some exceptions have been reported: *Artemisia dracunculus* L. (Asteraceae) EO was positive in rec-*Bacillus subtilis* assay (Zani et al., 1991). *Mentha spicata* L. (Lamiaceae), *A. graveolens, Pinus sylvestris* L. (Pinaceae) and *M. piperita* EOs were found to be genotoxic in different assays like the *Drosophila melanogaster* somatic mutations and recombination test (SMART) (Franzios et al., 1997; Karpouhtsis et al., 1998; Lazutka, JR et al., 2001); Anise EO, *trans*-anethol *e.g.* from fennel, *β*-asarone *e.g.* from *Acorus calamus* L. (Acoraceae), terpineol (*p*-menth-1-en-ol), *trans*-cinnamaldehyde, carvacrol, thymol and S (+)-carvone proved to be active in the AMES test (Nestmann and Lee, 1983; Hasheminejad and Caldwell, 1994; Gomes-Carneiro et al., 1998; Stammati et al., 1999). However, it appears questionable, whether concentrations, which showed harmful effects *in vitro*, may be reached *in vivo* upon proper application. Some of the phenylpropanoids are converted to epoxides in the liver, which can thus become mutagenic (Wink and Schimmer, 2010). Complementarily, it has been shown using yeast strains (*Saccharomyces cerevisiae*) *in vitro* that exposure to EOs can induce mitochondrial damage affecting mitochondrial membranes and DNA. This can lead to the formation of cytoplasmic petite mutants with respiratory deficits. The specific composition of an EO affected the rate of this induction, similar to cytotoxicity (Bakkali et al., 2005). In this context, special selections of plants are cultivated, like calamus (*A. calamus*), which are poor in *β*-asarone, for example (Bertea et al., 2005).

It can therefore be assumed that since most EOs have been shown to be cytotoxic but not mutagenic, it is likely that most of them are also non-carcinogenic. Nevertheless, some EO or some of their components can be considered as secondary carcinogens after metabolic activation. EOs like those from *Salvia sclarea* L. (Lamiaceae) and *Melaleuca quinquenervia* (Cav.) S.T.Blake (Myrtaceae) may cause estrogen-like secretions which may induce estrogen-dependent cancers (Cuba, 2001). Others (e.g., orange, lemon and *Litsea cubeba* (Lour.) Pers.; Lauraceae) may contain photosensitizing molecules, such as flavins, cyanins, porphyrins and hydrocarbons, and can cause skin erythema or even cancer (Kejlová et al., 2010). The photosensitizing furocoumarin psoralen found in some EOs is known to induce phototoxic effects and may induce skin irritation or cancer, like phytophotodermatitis, after formation of covalent DNA adducts when exposed to ultraviolet A or solar light (Averbeck et al., 1990; Averbeck and Averbeck, 1998; Nguyen et al., 2020). However, in the dark, the same oil is neither cytotoxic nor mutagenic by itself. So, there are EOs with phototoxic activities, non-phototoxic but cytotoxic activities *in vitro* (*Santalum spicatum* (R.Br.) A.DC. syn. *Fusanus spicatus* (Santalaceae) Australian wood EOs) and EOs with phototoxic and *in vitro* cytotoxic activities [*Citrus aurantium* subf. *dulcis* (Yu.Tanaka) M.Hiroe syn. *Citrus gracilis* subsp. *dulcis* (Rutaceae) and *C. citratus*; murine fibroblastic cell line 3T3 and rabbit cornea derived cell line SIRC; (Dijoux et al., 2006)]. Recently, it has been demonstrated that furanocoumarins may protect terpenes from oxidation (Bitterling et al., 2022a; 2022b). So, it appears that interactions between the single compounds of an EO as well as between different types of MOCS (for example EOs and polyphenols) may have been overlooked in the past and deserve closer examinations.

The relevance assessment of these data and their importance for *in vivo* experiments is generally difficult since they were mostly collected *in vitro* or for single compounds or in unphysiologically high concentrations.

## Therapeutic Uses of EOs

The structural diversity as well as multi-component character of EOs led to a high number of physiological targets and the usability in different indication areas. Furthermore, therapeutical options of EOs are given by the distinct types of application. Inhalations can reach the upper and lower respiratory tract (Sadgrove et al., 2021). Through topical application substances can reach different skin layers, muscles and joints. Gargle solutions reach the mucous membranes and capsules or teas transport EOs to the gastrointestinal tract and lead to systemic absorption (Seyyedi et al., 2014; Alshehri, 2018; Filipović et al., 2020). A unique and very interesting way to achieve therapeutic effects with EOs is via smelling as scent impulses reach different brain areas via the olfactory nerve (Agatonovic-Kustrin and Morton, 2018; Zhang and Yao, 2019; Chandharakool et al., 2020). Consequently, EOs are part of interesting therapy options in case of respiratory diseases, rheumatic disorders, inflammatory skin diseases, gastrointestinal complaints as well as sleep and mental illnesses. Nevertheless, in all therapeutic treatments, the primary goal is the achievement of maximum therapeutic benefit while minimizing toxic and other undesirable side effects. The selection of the right essential oil, its dosage, method of application and integration in daily routine is correspondingly complex.

For most EOs, there is currently not enough reliable information regarding the active EO compounds and their molecular targeting *in vitro* and *in vivo*, but also regarding the best dosage to achieve the optimal efficacy and to minimize undesirable side effects for optimal safety. In our point of view, there are two consecutive ways to fill the knowledge gaps.

First, the efficacy and safety of an EO is evaluated based on the knowledge of other EOs with comparable composition or similar molecular structures. Since each component of a mixture exhibits its individual pharmacokinetic profile, the metabolic fate of an EO is assessed based on its individual substances and their known properties. This procedure is accepted and applied for most EOs (Tisserand and Young, 2014). Second, the complexity of EOs should be considered more in their entirety. In MOCS mutual interactions of individual compounds as well as interactions with their molecular environment may occur, resulting in synergistic, additive, or antagonistic effects. The latter are probably more rare according to current data. Therefore, the assessment of a complex mixture based on its individual active principles will offer a first impression, but results may not be valid in all predicted assumptions in the living organism. Appropriate *in vitro* and *in vivo* methods should be developed and applied that evaluate a more complete picture of complex natural MOCS. In such studies, the different model parameters may be observed after testing/administration and compared to the assumed physiological effects of the individual compounds to evaluate the difference between single constituents and complex blends. The literature impressively shows numerous examples of the advantages of administering specific compounds as natural mixtures, including lower toxicity. For example, EOs or extracts with safrole showed the unexpected absence of genotoxicity and carcinogenicity when compared to the neat substance at the same concentration level (Ishidate et al., 1984; Bhide et al., 1991; Choudhary and Kale, 2002). The reduced acute toxicity in EOs with thymol is another prominent example (Karpouhtsis et al., 1998). Further investigations seem worthwhile to get an even more complete picture of the benefit of EOs as MOCS as compared to isolated compounds.

The specific composition of the EO, the method of administration, the dosage, the frequency, and the duration of administration influence bioavailability. In this context, the (relative) bioavailability of a substance is defined as the proportion of the administered dose that reaches systemic circulation in unchanged form. So, an intravenous administration of a substance is equivalent to 100% bioavailability (Koch-Weser, 1974; Atkinson, 2007). Three major routes of intake have been assessed for EOs: the respiratory tract (including the olfactory system), the gastrointestinal tract, and the skin/mucosa. Most studies report rapid absorption of lipophilic EOs components, following oral, dermal and inhalative, but also rectal and vaginal administration, nevertheless they differ with regard to ADME parameters and it is generally concluded that metabolic data on humans are still incomplete (Kohlert et al., 2000; Schmitt et al., 2009; Bach et al., 2021). Furthermore, bioavailability is highly individual, and the same substance can be metabolized differently in a collective despite choosing the same application route. This applies to quantitative (fast versus slow metabolizer) as well as qualitative aspects (different metabolic profiles). In addition, the same individual may process the same compound differently depending on food intake and comedication. Further factors are health status, diet, age, skin integrity, gut microbiota, and other metabolic variations that may depend, for example, on the time of day (chronobiology) (Hurst et al., 2007; Karalis et al., 2008; Zhang et al., 2021).

The most common routes of administration of EOs: Dermal/mucosal, inhalative (including olfactory system) and oral (via GI), will be discussed in more detail in the following.

### Dermal/Mucosal (Topical) Administration

The skin is the largest organ of the human body with a thickness of about 3 mm and a layered structure. It provides protection from external insults, regulates the body temperature, but also the exchange of water and other compounds, such as minerals, fats, and various compounds resulting from metabolic transformations (Schommer and Gallo, 2013; Dąbrowska et al., 2018). The skin consists of an outer epidermis and the underlying dermis. The stratum corneum is the most important protective layer of the epidermis, consisting of dead cells embedded in a lipid matrix. Below the living epidermal cells are formed in the deep epidermis. The dermis below the epidermis consists of nerves, sweat and sebaceous glands, hair follicles, blood and lymph vessels, followed by the subcutaneous tissue, mainly fat (Ng and Lau, 2015). This general structure is variable and can differ depending on the body region. Therefore, different parts of the body may respond differently to EOs. Theoretically, there are two pathways for EOs absorption: the intercellular pathway (between skin cells) and the transcellular pathway (through cells) (Michaels et al., 1975). A third possible route of entry is through the hair follicles, bypassing the stratum corneum (Scheuplein and Blank, 1971; Meidan et al., 2005; Herman and Herman, 2015). Components that have been absorbed into the skin can be stored in the epidermis for a period of up to 72 h and then enter the systemic circulation via the dermis and its blood capillaries. However, the majority is absorbed within 24 h (Chidgey and Caldwell, 1986; Beckley-Kartey et al., 1997).

The protective stratum corneum consists of hydrophilic and lipophilic regions, which means that strongly water-soluble molecules such as glucose diffuse poorly through the lipophilic regions, while strongly lipophilic substances such as cholesterol and many other terpenoids can hardly cross aqueous regions (Wester and Maibach, 2000). To permeate through the skin barrier, substances should possess lipophilic properties as well as a certain degree of water solubility to facilitate the passage from the dermis into the bloodstream (Wepierre et al., 1968; Chacko et al., 2020). Numerous EO constituents appear to enhance their own and other substances’ dermal absorption. For example, methyl salicylate may accomplish this in part by increasing local capillary blood flow and thus acting as a rubefaciens (Cross et al., 1999). Other compounds can temporarily alter the transport properties of the stratum corneum by interacting with intercellular lipids (Williams AC. and Barry BW., 1991; Williams A. C. and Barry, B. W. 1991). Carveol, *α*-pinene and terpinene-4-ol significantly boost permeation of water and ethanol in isolated human epidermis after 4 h (Magnusson et al., 1997). Also, (+)-limonene accelerates the transfer of citronellol and eugenol. Both *α*-pinene and *β*-myrcene similarly increase the permeation of phenylethanol (Schmitt et al., 2009). For rose oil components it was shown that all substances under investigation, except *α*-pinene and isomenthone, reveal skin permeation rates, which are several times higher when applied in rose oil as compared to the individual substance only (Schmitt et al., 2010). Cooperative interactions between EO constituents that promote the absorption of EOs and their own constituents may be the reason. EOs interact with lipids of the skin, reducing their highly ordered state and thus their barrier function, which facilitates passage through the dermis. Some terpenoids improve the transport properties of the skin in such an efficient manner that they are used intentionally to increase the absorption of various drugs. This should be considered when treating skin with EOs, as they may modulate the absorption of drugs already applied to the skin (Gao and Singh, 1998; Hasan and Farooqui, 2021). Apart from the chemical EO composition, the absorption of EOs through the skin is dependent on several further factors, such as temperature, hydration, pressure, specific skin condition, skin microbiome, and age (Buck, 2004; Schmitt et al., 2009; Schommer and Gallo, 2013; Herman and Herman, 2015). In the last decade, it has become particularly evident that the skin microbiome not only influences skin appearance and disease (e.g., acne) but is also linked to the gut microbiome and plays a major role in immune defense (Beri, 2018; Nakouti et al., 2022). EOs and other natural MOCS can be used to rebalance the skin microbiome and the resulting clinical picture, promoting a healthy skin microbiology (Han et al., 2017; Wallen-Russell and Wallen-Russell, 2017; Białoń et al., 2019; Bunse et al., 2022).

Three other routes of topical EO administration, to bypass the gastrointestinal tract or hepatic first pass metabolism exist, namely the sensitive mucosa of the mouth, rectum, and vagina. This kind of application allows that the EO compounds reach their target unaltered. It also represents a most efficient way to administer a remedy locally to the lower colon or to treat vulval and vaginal, as well as mouth infections or irritations. An adequate dose is crucial because all three tissues covered with mucous membranes are highly sensitive to irritation, especially if the EO is unevenly dispersed. For example, rectal administration of 1,8-cineole, menthol or thymol resulted in high, moderate and zero elimination via the lungs in rats, respectively (Grisk and Fischer, 1969). An EO preparation administered for the treatment of vulvovaginitis, such as tea tree and geranium oil reaches the target site in a direct way and can reduce and eliminate infectious causes and inflammation (Blackwell, 1991; Maruyama et al., 2008). Oral infections, postoperative wounds or bad breath caused by bacteria, as well as xerostomia can be treated with appropriate EOs in e.g., mouthwashes (Fischman et al., 2004; Alshehri, 2018; Scotti et al., 2018; Filipović et al., 2020). *Syzygium aromaticum* (L.) Merr. & L.M.Perry syn. *Eugenia caryophyllata* L. (Myrtaceae), *Mentha arvensis* L. (Lamiaceae), *Leptospermum scoparium* J.R.Forst. & G.Forst. (Myrtaceae), *Thymus capitatus* Cav. (Lamiaceae) and *Thymus vulgaris* L. (Lamiaceae) essential oils showed *in vitro* antibacterial activities on oral pathogenic bacteria (Tardugno et al., 2018). Thus, as an example, mouth rinses with chamomile (*Matricaria chamomilla* L.; Asteraceae) essential oils might be applied to treat oral mucosal lesions and alleviate the suffering of recurrent aphthous stomatitis (RAS) patients (Seyyedi et al., 2014; Salehi et al., 2019).

### Inhalative Administration

During inhalation, substances pass through the trachea into the bronchi and from there into the increasingly fine bronchioles and finally into the microscopic, sac-like alveoli of the lungs, where gas exchange with the blood mainly takes place. Components of EOs can be absorbed extremely efficiently into the bloodstream via the alveoli. Uptake depends on the speed of blood flow through the lungs, the rhythm and depth of respiration, and the specific lipophilicity of the molecules (Breuninger et al., 1970; Jaradat et al., 2016). EOs components that find their way into the bloodstream via inhalation can easily reach the central nervous system. So, caution should be taken with neurotoxic compounds. Previous studies on EOs that entered the bloodstream via inhalation showed no undesired effects, as the concentration of EOs or their substances hardly reached a dangerous level in the ambient air or in the body (Falk et al., 1990; Buchbauer et al., 1991; Jirovetz et al., 1992; Falk-Filipsson et al., 1993). The situation may be different with neurotoxic ingredients, such as pinocamphone or thujones. However, there is currently insufficient information available to define which constituents represent an inhalational risk. Furthermore, molecules and drugs can be absorbed through the olfactory epithelium and its membranes and enter the bloodstream (Kristensson and Olsson, 1971; Conway and Ghori, 2022). In addition, EOs can bind and modulate receptor proteins of the olfactory bulbs which can directly transmit signals via synapsis to electrochemical nerves and to the brain (Angelucci et al., 2014). More than 1000 different types of olfactory receptor genes are known for mammals, and less than 400 genes which play role in human olfactory system. EO individual compounds can interact with these receptors and thus affect behavior and physiological conditions. These effects can be used in aromatherapy (Firestein, 2001; Koyama and Heinbockel, 2020): Different EOs which have an effect on the psyche may be used to reduce anxiety, to treat sleep disorders and improve attention and memory performance (Lizarraga-Valderrama, 2021). Various studies showed that some of the EO substances interact with most neurotransmitter systems, *e.g*., in the limbic system (amygdala-hippocampal complex) by acting on different receptor proteins (López et al., 2017; Kennedy et al., 2018; Kontaris et al., 2020). Lavender oil is another prominent example, which acts on the serotonin 1A receptor (Baldinger et al., 2014).

### Oral Administration

Oral administration has several advantages: The patient can take the preparation him- or herself, the dosage is easier and often there is a high bioavailability. For example, following ingestion of capsules containing 1,8-cineol, limonene and *α*-pinene as most abundant compounds, a treatment of bronchitis and sinusitis, the bioavailability of the main constituent 1,8-cineole reached 95.6% (Zimmermann et al., 1995). When taken orally, higher doses can be applied, but also require greater care in dosing. However, it should also be noted that the absorption of substances from EOs can be modulated by the simultaneous intake of food or comedication (Kohlert et al., 2000; Zhang et al., 2016; Stevanović et al., 2018). Furthermore, EOs can provoke mucosal irritation in sensitive individuals (Fischman et al., 2004). Since irritation depends on the local concentration, EOs should never be taken undiluted, but in formulations *e.g*., with edible oil or encapsulated. In case of an overdose or an adverse reaction, nausea and vomiting of incorrectly administered EOs may occur (Woolf, 1999). Gastric digestive enzymes are capable of degrading and converting individual ingredients (Michiels et al., 2008; Stevanovic et al., 2020). For example, esters can be hydrolyzed in the stomach resulting in metabolites with altered physico-chemical properties and modulated absorption. After absorption of the substances into the bloodstream, they reach the liver, where a significant portion is converted in first-pass metabolism. However, a few compounds may also become toxic as a result (Hoskins, 1984; Wink and Schimmer, 2010; Zárybnický et al., 2018). Therefore, dosage, frequency of ingestion, patient age, medical history, and life circumstances are discussable circumstances to successful oral administration of EOs, and self-medication without medical or pharmaceutical supervision is not advised. Rather, the potential of EOs can only fully be exploited when applied with knowledge and care.

## Metabolism

In the body, a large proportion of externally supplied compounds are metabolized for example by the gut microbiome (Bento et al., 2013). A compound may be converted into one or more different metabolites, with altered physical, chemical and biological properties. During this process, the metabolite usually becomes more hydrophilic than its parent compound and can thus be excreted more quickly via the kidneys. Furthermore, excretion routes are the skin and the respiratory tract (Brown, 1985; Heaney et al., 2016; Elpa et al., 2021). The liver is the most important metabolizing organ for EO compounds, but the skin, nervous tissue, kidneys, lungs, intestinal mucosa, blood plasma, adrenal glands and placenta also show metabolic capacities (Gropper and Smith, 2012). As EOs are MOCS each component has its own metabolic fate, therefore, it is very complex to specify the metabolism of an EO not only *in vitro* but especially *in vivo*. Typically, a compound undergoes multiple stages of transformation, and each constituent is eliminated from the body by one or more pathways with specific kinetics (Kohlert et al., 2000).

In phase 1 reactions, particularly reactive functional groups undergo changes such as hydrolysis, for example by non-specific esterases. Or, cinnamic acid methyl ester is hydrolyzed, thus releasing cinnamic acid and methanol and salicylic acid methyl ester is transformed into salicylic acid and methanol (Gibbs, 1908; Davison et al., 1961; Fahelbum and James, 1977). In addition, reactions such as oxidation, *e.g*., by cytochrome P_450_ enzymes, and reduction are important mechanisms of metabolization of EO compounds (Michaels et al., 1975; Miyazawa and Haigou, 2011). In phase 2 reactions (conjunction reactions) substances are covalently bound to polar endogenous molecules, to substantially reduce their lipophilicity and facilitate their excretion. Most drug and EO constituents undergo reactions of this type (Bowman et al., 1982; Miyazawa et al., 2002). This includes glucuronidation, sulfation, and glutathione conjugation, whereby the first is the most common phase 2 reaction in mammals for detoxifying foreign substances (Dutton, 2019; Jäger and Höferl, 2020). Interestingly, recent results showed that phase 2 metabolism of phenolic compounds and terpenoids is significantly more complex than previously thought. Depending on the concentration and specific structural elements different metabolism via sulfotransferases and glucuronic acid transferases take place (Tremmel et al., 2021). Also, the excretion route seems to play a significant role. Oral intake of a thyme extract to humans led to the detection of thymol sulfate in plasma and urine, whereas thymol glucuronide was only present in urine (Kohlert et al., 2002). In contrast, for L-menthol a glucuronic acid conjugate was observed in humans as the main metabolite in plasma and urine (Hiki et al., 2011).

The liver plays a central role in the metabolism of EOs. Some EO components have been reported to alter the production and activity of drug metabolizing enzymes. In particular, these responses have been reported for enzymes of the cytochrome P_450_ family (Zehetner et al., 2019). In many cases, enzyme induction results in decreased rather than increased toxicity because the toxic chemicals are more readily eliminated. For example, eugenol can increase the activities of specific liver enzymes when administered to rats, and linalool increases the activity of cytochrome *b*5 (Parke et al., 1974; Rompelberg et al., 1993; Zehetner et al., 2019), which forms part of the respiratory enzyme chain. In all cases relatively high doses were administered by either oral or intraperitoneal injection routes and it seems highly unlikely that humans would be exposed to equivalent amounts of EOs under physiological conditions. Therefore, it can be assumed that EOs do not pose a significant risk of affecting blood levels in humans by cytochrome P_450_ induction when applied topically or orally.

## Interactions Between EO Compounds – Safety Assessment

Upon EOs application, interactions may occur between one or more of its ingredients, as well as with matrix compounds and individual active pharmaceutical ingredients or food components. Often, the major constituents reflect quite well the biophysical and biological properties of the EOs from which they were isolated, with the extent of their effects mainly depending on their respective concentration when tested alone or in EOs (Ipek et al., 2005). Such interactions are difficult to predict but may be categorized. The simplest is additivity, where the effects and potency of the mixture are as predicted by the known effects and amounts of its ingredients, *i.e.,* there is no mutual influence of individual compound properties. The second possibility is synergy (synergism, potentiation), which means the effect of the mixture is significantly increased. Different molecules and their active groups thus enhance their properties when applied in combination. Nevertheless, possible synergism between individual compounds of an EO is complex and cannot be limited exclusively to a few major constituents (Ronzheimer et al., 2022; Schreiner et al., 2022). Thirdly, antagonistic effects may be observed which is the opposite of synergy. This means the different ingredients of a mixture or when two substances are administered causes a weakening of the effect compared to what is expected from the individual compounds (Chou, 2006; Elshafie and Camele, 2016; Połeć et al., 2022). The fourth effect described for EOs components is cooperative interaction. For example, limonene can enhance the permeation of citronellol and eugenol in human skin epidermis (*in vitro*) (Schmitt et al., 2009).

## Preclinical Data

As an example, investigation into the biological activity of linalyl acetate, terpineol, and (±)-camphor individually or in combination against human colon cancer cell lines *in vitro* demonstrated a synergistic mode of the constituents in the mixture. Neither camphor nor terpineol alone had any effect or activity and that of linalyl acetate was only marginal. Together with terpineol, the activity was increased to moderate (33 and 45% reduction in proliferation, respectively; concentration 10^–3^ M each). However, when all three substances were used together in the natural blend, proliferation in the two human cancer cell lines HCT-116 (p53^+/+^ and p53^−/−^) was reduced by 50 and 64% (concentration 10^–3^ M each), respectively. No toxic effect on normal intestinal cells was reported (Itani et al., 2008).

The lower toxicity of carvacrol in the presence of thymol is an example of antagonistic action (Karpouhtsis et al., 1998). In thyme oil, high levels of thymol and/or carvacrol, totaling 31%–80% thymol and carvacrol, were found to be associated with antagonistic properties. In feeding studies with rats, the acute oral toxicity (LD_50_ values) of these two compounds were 980 mg/kg BW and 810 mg/kg BW, respectively. Assuming an average LD_50_ of 895 mg/kg BW each, the LD_50_ of a thymol/carvacrol thyme oil would range from 1.1 to 2.9 mg/kg BW. In fact, the oral LD_50_ for this thyme oil in the feeding study was 4,700 mg/kg BW, which is about half as toxic as the thymol and carvacrol content would suggest. Interactions with further EO compounds naturally present in the oil could of course not be excluded. However, this influence of only two compounds in combination alone shows how complex a biological effect in MOCS may be composed. Further data are reported for antagonism in skin sensitization, which is known as quenching. In cinnamaldehyde-sensitive subjects, a quenching effect on sensitization by cinnamaldehyde was shown for (+)-limonene in three of 11 human subjects, and in combination with eugenol, a quenching effect was shown in seven of the same 11 subjects. It has been postulated that this may be due to competitive inhibition at the receptor level (Guin et al., 1984). To confirm this assumption, further studies should follow.

EOs contain complex mixtures of substances that may be harmful and/or protective. Plants use them in order to protect themselves against reactive oxygen species (ROS) produced automatically during the process of photosynthesis. Especially phenolic EO constituents, such as thymol, have antioxidant properties. Such properties of these molecules can mediate reduced toxicity, such as attenuation of phototoxicity, allergenicity or mutagenicity. This is evident *e.g.*, for carvacrol, thymol, and eugenol and their antihepatotoxic effects (Jiménez et al., 1993; Kumaravelu et al., 1995), for 1,8-cineole and its gastro protective effect (Santos et al., 2001), for thymoquinone and its antinephrotoxic action (Badary, 1999) and for linalool and its antimutagenic action (Berić et al., 2008).

The quality of these effects may be considered either antidotal to possible toxicity or simply therapeutic, *e.g.*, the antispasmodic effect of anise oil from *P. anisum* or cumin oil from *Cuminum cyminum* L. of the Apiaceae family (Pourgholami et al., 1999; Sayyah et al., 2002), the anti-asthmatic action of turmeric oil (*C. longa*), may chang oil from *L. cubeba* (Venugopal and Dhanish, 2018; Smruti, 2021) and the anticarcinogenic action of (+)-limonene and perillic acid in skin cancer (Lluria-Prevatt et al., 2002; Raphael and Kuttan, 2003). Biological properties of a mixture can thus be enhanced or attenuated by its constituents. So, the presence of large amounts of antioxidant, antimutagenic, and anticarcinogenic constituents in EO, which contains low amounts of carcinogens, may render this oil harmless. In that sense, for biological purposes, it is more informative to study the entire oil containing assigned single effective substances, rather than only single components, because the concept of interactions appears to be more meaningful for therapeutic purposes. In addition, other minor components can modulate the activity of the main compounds. (Franzios et al., 1997; Santana-Rios et al., 2001; Hoet et al., 2006).

## Therapeutic Potentials of MOCS in Humans

As mentioned above essential oils consist of a plethora of different secondary metabolites from various metabolic pathways. The biological activity of these complex mixtures has hardly been investigated in its entirety on a molecular level. Nevertheless, the available methods are not designed for such diverse MOCS, their specific multi-activities can currently only be assessed more precisely based on their known individual compounds. A look at the known modes of action of secondary plant constituents may help to provide a more complete picture of EOs.

### Interactions With Biomembranes

Biomembranes are barriers of eukaryotic and prokaryotic cells which separate the cells from the environment and compartmentalize specific metabolic entities or cell organelles like mitochondria, chloroplasts etc. They consist of a semi-liquid double layer, which is mainly built up by phospholipids, glycolipids, and cholesterol. Membrane proteins, ionic channels, receptors, transporters, and carbohydrates are also incorporated or attached. The most important tasks of biomembranes comprise the transport of substances, the communication, and the exchange of substances with other cells and tissues (Gennis, 1989; Bondar, 2019). Lipophilic secondary plant compounds like EO constituents can interact with the biomembrane, *e.g.*, by attachment or incorporation. For example, carvacrol, *p*-cymene, thymol and *γ*-terpinene may act as substitutional impurities forming gross perturbation of the lipophilic fraction of the plasma membrane of microorganisms (Cristani et al., 2007). Further studies showed that *β*-caryophyllene and *β*-caryophyllene oxide are able to interact with the phospholipid bilayers (*in vitro* biomembrane model of dimyristoylphosphatidylcholine multilamellar vesicles; Sarpietro et al., 2015). This can lead to altered membrane fluidity and increased permeability. Some plant constituents may also modulate ion channel activity, such as mint oil affecting calcium channels and intestinal smooth muscle cell motility. Adenosine transport in endothelial cells is also inhibited by some essential oils, which may be associated with spasmolytic and local anesthetic effects (Melzig and Teuscher, 1991).

Disruption or lysis of the biomembrane usually leads to necrotic cell death. This mechanism could be found for some EOs with antibacterial activities (van Wyk, 2015; van Wyk and Wink, 2015, 2017). The first inherent step of most EOs, and thus their ability to interact with multiple targets, is to cross membranes.

### Modification of Proteins

Due to their diversity of carbon skeletons in combination with a multitude of functional groups (*e.g*., aldehyde group, SH group, epoxide group, double bond, triple bond, etc.; Figure 3), originating from evolution processes, plant secondary metabolites exhibit diverse chemical structures/variations with different chemical and physiological properties (Polya, 2003; Teuscher and Lindequist, 2010; Wink, 2010; Velu et al., 2018). Several of these functionalities, especially unsaturated carbonyls, may build covalent bonds with proteins, peptides, but also DNA (Wink, 2008; Wink, 2012). Furthermore, aldehydes can form amides or imines with amino groups of proteins, amino acids, or DNA bases. Epoxides react with amino and SH groups of proteins as well as DNA bases. Isothiocyanates bind to amino or SH groups and allicin (from garlic) or exocyclic methylene groups (*e.g.* in sesquiterpene lactones) can bind to SH groups and glutathione (Wink, 2015). The modifications usually target cell proteins, such as enzymes, receptors, transcription factors, ion channels, transport, or cytoskeletal proteins. Thus, the protein conformation may be modified resulting in altered receptor binding affinity, protein-protein recognition, catalytic activity, etc. This also includes proteins involved in diseases, like Creutzfeldt–Jakob disease, Gerstmann–Sträussler syndrome or Alzheimer’s disease, and possibly some more, which are unknown to date (Palmer et al., 1991; Karakaya et al., 2019). In addition, reactions between proteins and oxidized polyphenols may decrease allergenicity, which was shown for apple fruits (Siekierzynska et al., 2021). The same protective mechanisms are likely found in other multi-target-actions of natural MOCS, such as EOs.

Also, phenolic compounds from EOs may influence proteins and peptides (Figure 3) with their hydroxyl groups by forming hydrogen bonds. Furthermore, phenolic OH groups can dissociate, resulting in phenolate ions under physiological conditions, which easily form ionic bonds to positively charged ammonium groups of amino acids (*e.g*., lysine, arginine) (Wink, 2005, 2008; Wink, 2012; van Wyk, 2015; van Wyk and Wink, 2015, 2017). If numerous hydrogen and ion bonds are formed with a protein or its functional units, conformation and thus also the functionality of the protein will be modified. If transcription factors are affected, gene regulation is altered as well (Pakalapati et al., 2009; Holtrup et al., 2011; El-Readi et al., 2013).

### Interactions With Nucleic Acids

Due to their diverse functionality, plant secondary metabolites can also intercalate or alkylate DNA, which can lead to mutations and even cancer. Wink also described, that important alkylating secondary metabolites are pyrrolizidine alkaloids in the Boraginaceae and some Asteraceae representatives, aristolochic acids in Aristolochiaceae, furanocoumarins in the Apiaceae, and ptaquiloside in the fern *Pteridium aquilinum* (L.) Kuhn (Dennstaeditiaceae) as well as cycasine in Cycadaceae (Wink and Schimmer, 2010; El-Shazly and Wink, 2014; van Wyk, 2015; van Wyk and Wink, 2015, 2017). EOs have also been reported to interact with nucleic acids or associated enzymes of viruses and inhibit their replication, which might offer promising therapeutic opportunities in the treatment of influenza or COVID-19 (Asif et al., 2020; Da Silva et al., 2020; Panikar et al., 2021; Wani et al., 2021).

### Antioxidant Properties

Reactive oxygen species (ROS), which inevitably occur in plant cells during photosynthesis, can alter functional proteins, lipids and nucleic acids. ROS can oxidize the DNA base guanosine to 8-oxoguanosine. While guanosine would normally pair with cytosine, 8-oxoguanosine no longer pairs with cytosine but with adenosine. This will lead to mutations. ROS can lead to cell damage in the plant, which protect themselves against oxygen radicals by biosynthesizing antioxidant molecules such as phenolics and terpenoids. Under physiological conditions, the formation of ROS also occurs in human tissues.

In the case of long-term oxidative stress, an overdose of ROS can lead to various health disorders, usually chronic, such as diabetes, metabolic syndrome, cardiovascular disease, and even cancer (due to DNA mutations). Medicinal plants, herbal medicines and products derived from algae, which are rich in polyphenols, often exhibit antioxidant effects in addition to other pharmacological activities and therefore may prevent and help to cure disorders (van Wyk, 2015; van Wyk and Wink, 2015, 2017; Wink, 2022).

As about 22,000 isoprenoids and more than 100,000 of other secondary metabolites are known, the studies carried out so far can only be the beginning to exploit the therapeutic potential of MOCS in general and that of EOs in particular. The multi-component nature of natural extracts like EOs, results in an almost inexhaustible pharmacological toolbox with versatile modes of action (Schreiner et al., 2021). The multi-target sites of action of EOs are due to their nature being mixtures. The mutual physico-chemical interactions with molecular targets but also with the tissues they pass through offer the possibility of a complex effect that cannot be reached with single compounds. This versatility gives hope that EOs and other MOCS might play a central role in combating modern health challenges.

One of these future challenges, *i.e*., the antibiotic resistance will be discussed here in more detail.

## Antibiotic Resistance and EOs as Potential Answers

Antibiotic resistance is a major and growing problem in health care and livestock farming. Resistance phenomena arising from mutation are common among pathogenic bacteria. The molecular and structural determinants underlying resistance towards the major antibiotic classes are as diverse as nature. Most hitherto identified mutations leading to antibiotic resistances can be categorized into target modification, drug inactivation, and drug transport (efflux). Recent research on the development of resistance also suggests that changes in the metabolic pathway of bacteria, including mutations of the relevant genes, may lead to possible antibiotic resistance (Lopatkin et al., 2021). Since changes in individual proteins are the underlying principle of most of these resistance mechanisms, natural MOCS like EOs offer a great potential for overcoming multidrug-resistant infections through their multi-target properties. This antimicrobial potential of EOs for human and animal health is an evolutionary side effect due to plant interaction and defense against various pathogens.

### Synergistic Actions Between EOs and Antibiotics

Since the extensive use of antibiotics from 1945 onwards, bacteria have increasingly been selected for resistance to single or multiple (multi-resistance) antibiotics. All over the world, people are aware of the increasing ineffectiveness of these antibiotics and are intensively searching for novel active substances and new targets. Therapeutically used natural derived antibiotics are usually produced by fungi and bacteria, but plants have successfully developed multi-component-based strategies to defend themselves against germs for thousands of years. Thus, it is a promising challenge in the future to develop multi-component-based antibiotic strategies adapted from nature, or at least consist of a combination of MOCS and conventional antibiotics. Studies on EOs and their ingredients in combination with known antibiotic drugs show promising interactions (Tables 1, 2). For example, synergism may occur when different compounds simultaneously attack different sites of a bacterial cell (multi-target effect). Alternatively, there may be pharmacokinetic or physicochemical interactions, such as enhancement of solubility or bioavailability. The most commonly reported test method for assessing interactions with antibiotics is the checkerboard assay with calculation of the FIC (fractional inhibitory concentration) index (Hemaiswarya et al., 2008; Wagner and Ulrich-Merzenich, 2009).

**TABLE 1:** *In vitro* synergistic action between EOs and antibiotics. Methods used: Checkerboard assay (isobologram, fractional inhibitory concentration (FIC) index) or time-kill assay or fold reduction in minimum inhibitory concentration (MIC) or change in inhibition zone in the presence of EO vapor. [Adapted and compiled from Langeveld et al. (2014)].

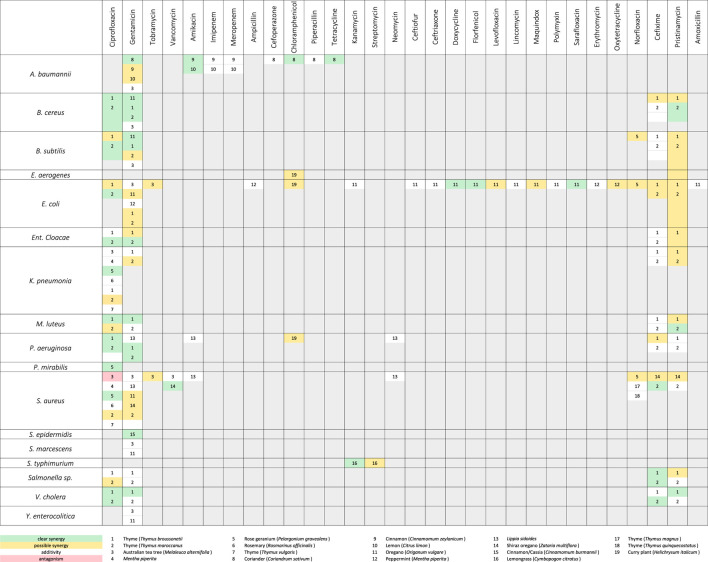

**TABLE 2:** *In vitro* synergistic action between EO constituents and antibiotics. Methods used: Checkerboard assay (isobologram, fractional inhibitory concentration (FIC) index) or time-kill assay or fold reduction in minimum inhibitory concentration (MIC) or change in inhibition zone in the presence of EO vapor. [Adapted and compiled from Langeveld et al. (2014)].

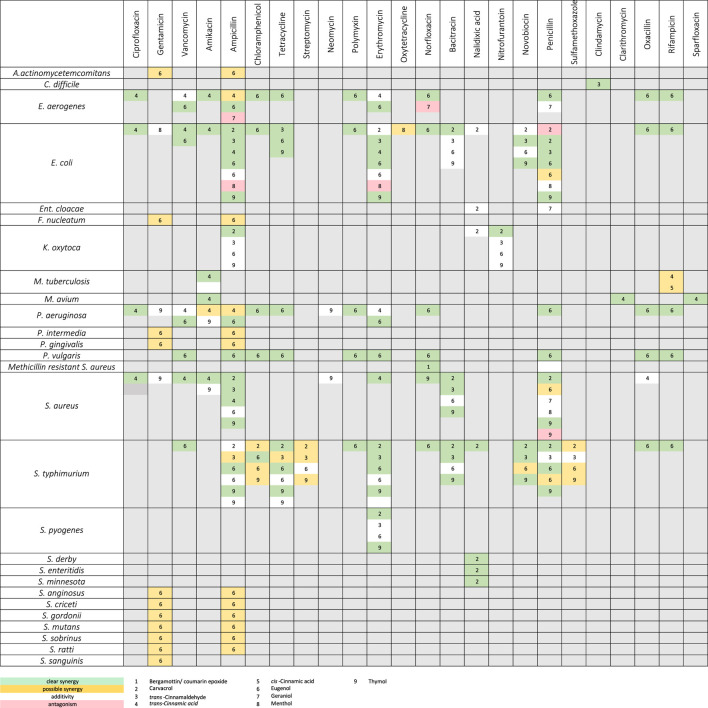

Synergistic effects of antibacterial agents in combination with antibiotics on different targets are thought to be most efficient. Also, it has been shown that *in vitro* synergism between EOs or EO ingredients and beta-lactam antibiotics do occur, as EOs often act on cell membranes whereas beta lactams target the cell wall. Oregano oil showed synergistic effects in combination with fluoroquinolones, doxycycline, lincomycin, maquindox or florfenicol against extended-spectrum *β*-lactamase (ESBL)-producing *Escherichia coli* (Si et al., 2008). As described before, MOCS are highly complex mixtures and their effects are based on the interaction of their individual compounds both on a quantitative and qualitative level. If individual ingredients are lacking or are present in an altered ratio, changes in the overall properties of an EO may occur. Therefore, basic research on naturally derived extracts (MOCS), on preparations derived therefrom, and derived pharmaceutical applications is an important basis to understand and apply the mechanisms of action appropriately. Consequently, an exact phytochemical characterization of MOCS is an always indispensable prerequisite. It is interesting to note that synergistic effects through pharmacokinetic and physicochemical actions have also been observed for secondary metabolites that do not possess pharmacological activity themselves. *p*-Cymene is such an ingredient which, in combination with carvacrol, improves its activity against *B. cereus*, presumably by accumulation in the bacterial membrane, thus modifying its structure and barrier function (Ultee et al., 2002).

Further *in vitro* synergism was monitored between oregano oil and doxycycline, florfenicol, or sarafloxacin against ESBL-producing *E. coli* from chickens (Si et al., 2008). Furthermore, oregano oil showed *in vitro* synergistic effects with gentamicin against *B. cereus*, *B. subtilis*, and a strain of *Staphylococcus aureus* (Rosato et al., 2010). In contrast, the combination with gentamicin was less effective (rather additive than synergistic) against *E. coli*, *Acinetobacter baumannii*, and another strain of *S. aureus*; the isobologram method showed some synergism, while the FIC index indicated an additive effect (Rosato et al., 2010). The combination of oregano oil and gentamicin only yielded an additive *in vitro* effect against *Yersinia enterocolitica* (Rosato et al., 2010). The combination of oregano oil with the antibiotics levofloxacin and maquindox against *E. coli* revealed low synergism (FIC index 0.5) (Si et al., 2008). A study on thyme oil showed synergistic *in vitro* effects against *S. aureus* and *Klebsiella pneumoniae* when applied in combination with ciprofloxacin (van Vuuren et al., 2009). The EO of Shiraz thyme (*Zataria multiflora* Boiss.) demonstrated synergistic action with vancomycin against methicillin-sensitive *S. aureus* (MSSA) and 12 clinical methicillin-resistant *S. aureus* (MRSA) isolates, although FIC data for individual strains were not reported (Mahboubi and Bidgoli, 2010). Vancomycin is among the few antibiotics available to treat MRSA infections, and yet resistance has already been reported according to Mahboubi and Bidgoli (2010). The composition of this Shiraz thyme EO (thymol 39%, carvacrol 15% and *p*-cymene 10%) is similar to oregano oils (Burt, 2004; Mahboubi and Bidgoli, 2010) and thus may offer a solution to possibly bypass vancomycin resistances and reduce antibiotic use.

Essential oils from cloves (*S. aromaticum*) and cinnamon (*Cinnamomum verum J.Presl;* Lauraceae) combined with lysozyme amplify the effects of a carbapenem- (imipenem) and an aminoglycoside-antibiotic (gentamicin) against the bacterial pathogens *Pseudomonas aeruginosa* and *Klebsiella pneumoniae*. The results indicate that the essential oils of both plant species reduce the minimum inhibitory concentrations of gentamicin and imipenem against multi-drug resistant clinical isolates of the two Gram-negative bacterial species and thus significantly increase the antibiotic effects (Sakr et al., 2021).

In addition, Australian tea tree oil (*M. alternifolia*) was studied *in vitro* in combination with aminoglycoside antibiotics. This revealed synergistic effects when treating *E. coli*, *Y. enterocolitica*, *Serratia marcescens*, and *S. aureus* with the EO and the antibiotic gentamicin (Rosato et al., 2010). When applying this combination against *A. baumannii*, *B. subtilis*, and a further strain of *S. aureus*, the FIC index was in the borderline range between additivity and synergism. Furthermore, tea tree oil combined with tobramycin also showed a synergism against *E. coli* and *S. aureus* (D'Arrigo et al., 2010). The mechanism is characterized by a multi-target effect because the aminoglycosides inhibited protein biosynthesis and tea tree oil damaged the cytoplasmic membrane of the bacteria. In contrast, tea tree oil has been shown to exhibit additive/undifferentiated activity *in vitro* with the glycopeptide vancomycin to control a clinical MRSA isolate, and antagonistic activity together with ciprofloxacin (LaPlante, 2007).

An overview of possible *in vitro* interactions of further antibiotics with EOs (Table 1) shows that there are still many gaps (empty fields) to be filled in this area of research. The need for research in this direction could be forward-looking. In addition, *in vivo* studies should be performed that investigate the combination of intravenously administered antibiotics with orally administered EOs.

### Interrelation Between Individual EOs Constituents and Antibiotics

Most studies on the interaction of EO ingredients and antibiotics have been performed *in vitro* and the underlying mechanisms have not yet been further investigated. However, since antibiotics specifically focus on one target and EO metabolites attack diverse sites of bacterial cells, it can be assumed that multi-target effects are working in most cases. However, a few effects can also be attributed to synergisms between antibiotics and EO constituents (Table 2) that target bacterial resistance mechanisms, such as inhibition of efflux pumps (Shahverdi et al., 2007; Lorenzi et al., 2009; Johny et al., 2010), which may also be due to membrane damage and metabolic disruption (Gibbons, 2008).

The EO constituent eugenol was tested *in vitro* in combination with antibiotics from eight different groups against the bacteria *E. coli*, *Enterobacter aerogenes*, *Proteus vulgaris*, *P. aeruginosa* and *S. typhimurium*. Synergistic effects were found among all antibiotics. These were most evident with ampicillin, polymyxin B, norfloxacin, tetracycline, rifampicin, and vancomycin, but synergisms were also detected in combination with penicillin and chloramphenicol (Hemaiswarya and Doble, 2009). Noteworthy, carvacrol and thymol seem to show stronger effects on antibiotic-resistant *S. typhimurium*, *E. coli* and *S. aureus* strains compared to eugenol. The mechanism has been suggested to be increased by antibiotic penetration across permeabilized membranes and/or inhibition of protective enzymes (Palaniappan and Holley, 2010). In fact, even minor differences in the molecular structure of EO constituents can have a significant impact on their ability to synergize with antibiotics. For example, carvacrol and thymol are structural isomers and only differ in the position of their hydroxyl group. While, carvacrol was found to act synergistically against *Klebsiella oxytoca* in combination with ampicillin and nitrofurantoin, thymol was indifferent (Zhang et al., 2011). In this regard, a benzene ring with prop-2-enal side group appears to be less synergistically active than methylethyl- and methyl side groups (Palaniappan and Holley, 2010; Zhang et al., 2011).

The results show that EOs may contain quite effective single compounds. Nevertheless, the efficacy can neither be attributed to these mono-substances alone nor to the quantitatively leading compounds. Rather, the mixture should be assessed in its complex overall composition and the understanding of possible mechanisms of action should be approached with the help of individual compounds. The study results of studies on individual compounds show how precisely adjusted the compounds in their respective composition may act and justify the assumed multi-target mechanisms of action. Of course, it will be necessary to adapt existing methods for the investigation of complex mixtures or to develop completely new experimental procedures to meet the requirements of complexity (Ronzheimer et al., 2022; Schreiner et al., 2022). Our current scientific techniques seem to have reached a limit.

## Concluding Aspects for Future Research

The long-term use of plant derived EOs, but also other naturally derived MOCS, does not come by chance. As the history of our medicine impressively demonstrates, medicinal plants, extracts, and formulations therefrom, are the basis of many pharmaceutical achievements of modern times. It seems to be true that “There’s an herb for every ailment”, or better that “there is a MOCS or single compound from nature” for every ailment.

With the help of new technologies, the extraction and processing of herbal preparations and the active ingredients derived therefrom have been improved and several single compounds have been discovered as leads. Whereas the search for single compounds from nature is still ongoing, the research on MOCS has stalled due to lacking experimental approaches limiting the look on the entire repertoire of the toolbox.

MOCS, using EOs as an example, impressively demonstrate how diverse and valuable complex mixtures are. With the help of their uncountable molecular structures and functional groups, they possess mechanisms of action, both known and unknown to date, whose chemical properties can complement and potentiate each other in the form of synergisms, antagonistically neutralize toxic effects or additively contribute to a stable basic structure.

In the case of EOs, the individual compounds not only seem to interact with each other, they also interact with their environment and can thus influence the activity or conformation of molecular targets, often proteins. At the same time, they can bind to receptors and trigger physiological and psychological reactions. These biological activities are reflected in the pharmacological and therapeutic effect of EOs as MOCS. In addition, EOs as natural mixtures can achieve a characteristic multifaceted effect, which, compared to an isolated compound, has not only one site of action, but multi-targets. MOCS thus do not only use one target, but likely the entire toolbox. These multi-target properties of EOs could for example positively influence the successful treatment of infections due to (multi-)resistant bacteria and thus help save lives. Studies have shown that natural EOs in combination with antibiotics are quite capable of enhancing the antibiotic effect. This could minimize or prevent the careless use of antibiotics and the associated selection of resistant bacteria. When complex EOs are used against pathogenic bacteria, the bacterium is attacked at many different sites simultaneously, which means that it usually cannot develop a targeted resistance mechanism and can thus be treated successfully. Homolog mechanisms have likely long been used in the plant kingdom for resistance to pathogens and have evolved steadily in evolutionary terms.

It is worthwhile to broaden our view and to integrate data from studies on the effectiveness of single, isolated compounds as individual pieces of the puzzle into the overall picture of complex effectiveness. Humans themselves and the nature around them consist of myriad of substances and a complete reduction to a few compounds does not seem to do justice to the real picture. To date, it has not been fully understood how drugs interact and react with endogenous enzymes, the variable human microbiome, foods, feeds, chronobiologic factors, ethnic-, age- and gender-specific characteristics, or even with other drugs. Much more research is needed in this area, and the complexity of possible interactions seemingly pushes our current methods to their limits. Therefore, new methods and techniques need to be developed and existing ones improved. The goal of helping people recover in the best possible way should be at the forefront. The benefits of phytotherapy using EOs and other MOCS, which have been approved for thousands of years, should not be forgotten, or underestimated. There is a great chance not only for phytochemical and pharmacological achievements, but also for the development of new methods for the evaluation of complex natural mixtures in connection with biological processes, for future, sustainable and affordable healthy therapeutic strategies.
